# Why Cognitive Sciences Do Not Prove That Free Will Is an Epiphenomenon

**DOI:** 10.3389/fpsyg.2019.00326

**Published:** 2019-02-26

**Authors:** Andrea Lavazza

**Affiliations:** Centro Universitario Internazionale, Arezzo, Italy

**Keywords:** situationism, determinism, conscious control, Libet, empirical free will

## Abstract

Is epiphenomenalism virtually entailed by the current empirical knowledge about how the mind/brain causes human behavior? I'll address this question by highlighting that recent discoveries in empirical psychology and neuroscience actually do not strike the final blow to the notions of free will and intentional agency. Indeed, most of the experiments that purport to show that our behavior is unconscious and automatic do not prove that it is indeed the case and that therefore we do not have free will. There are many reasons for this, including the fact that those experiments focus on a specific range of our behavior, one that manifests a significant correlation between unconscious priming and decisions or reactions. However, this doesn't mean that the entire range of our relevant behavior works the same way. It can be argued that there are situations of higher relevance in which we are fully conscious of our decisions or, at least, there are decisions such that psychological experiments cannot prove them to always be unconscious and automatic. However, the epiphenomenalist challenge may suggest that we should abandon some of the suppositions implied by a traditional idea of free will.

## Free Will: Definitions and Levels of Explanation

In most ages and cultures, free will has been considered a characteristic or capacity that human beings are generally endowed with and that has a special, if not unique, value (Van Inwagen, 1983). It was usually thought that the intrinsic freedom of individuals, distinct from the social and political one, was a prerequisite for dignity and moral responsibility (McKenna and Pereboom, 2016). Lay people generally think they have an intuitive idea of what free will is. However, scholars who have reflected on the topic from different perspectives have not agreed on a single definition of it, nor on necessary and sufficient conditions to exercise it. Moreover, philosophy has always raised the doubt that we might believe to be free even if we are not. Many thinkers, indeed, believe that the determinism we find in the physical world seems to be incompatible with freedom in the sense implied by free will.

Recently, science has brought new empirical evidence to support the thesis of the illusory nature of free will. And there is also a line of philosophical and political reflection that expresses skeptical optimism about free will (Pereboom, 2001, 2013; Caruso, 2012, 2013). According to these authors, the data at our disposal show that free will is an illusion, but this does not affect our lives (either individually or in society), because we can indeed do without the idea of free will and still defend ourselves against wrongdoers and reward the best individuals in the various fields of human activity, while reducing anger, resentment, and exasperated competition (Waller, 2011). However, there are reasons to doubt the groundedness of this perspective, whose undesirable consequences should not be underestimated (Lavazza, 2017a).

In this framework, which marks a break with respect to the past, the greatest challenge to realism about free will seems to come from epiphenomenalism. First of all, it might be useful to look at the terms under discussion, while stressing once again that there is no shared agreement on the definitions and, consequently, often philosophers and scientists end up talking about different things in the debate on free will. Once the scope has been circumscribed, we will see why epiphenomenalism is a greater challenge than classical determinism. Then, in the main part of the article, I will explain why not even epiphenomenalism seems able to bring decisive evidence to support the thesis that free will is an illusion.

In order to discuss the impact of epiphenomenalism on the idea of free will it is first necessary to define the key concepts. As mentioned, there is no universally shared definition of free will. According to a minimal definition, free will is “the variety of control distinctively required for agents to be morally responsible” (Vargas, 2011). Free will can also be more precisely defined by three conditions (cf. Walter, 2001). The first one is *the ability to do otherwise*. This is an intuitive concept: to be free, one has to have at least two alternatives or courses of action between which to choose. If one has an involuntary spasm of the mouth, for example, one is not in the position to choose whether to twist one's mouth or not. The second condition is *the control over one's choices*. The person who acts must be the same who decides what to do. To be granted free will, one must be the author of one's choices, without the interference of people and of mechanisms outside of one's reach. This is what we call agency, that is, being and feeling like the “owner” of one's decisions and actions. The third condition is *the responsiveness to reasons*: a decision can't be free if it is the effect of a random choice, but it must be rationally motivated. If I roll a dice to decide whom to marry, my choice cannot be said to be free, even though I will freely choose to say “I do.” On the contrary, if I choose to marry a specific person for their ideas and my deep love for them, then my decision will be free (Lavazza, 2016).

This is a very thick definition of free will, with very demanding conditions. It borders on the idea of Ultimate Authorship, which however captures all the traditional insights and reflections on freedom understood in the “metaphysical” sense. From here it is possible to restrict the scope of free will to a thinner definition, one that is also suitable for the scientific data emerging from the laboratories. In fact, the idea of free will could be summarized in, and circumscribed to, that of “conscious control” on one's choices and decisions, where the qualification of “conscious” does not entail constant and relentless behavioral control but can also rely on habits or brain processes triggered at a time prior to the exercise of control. Even though this definition is unlikely to find general consensus, it could still be a good starting point.

Whatever idea of free will we may consider, physical determinism has always presented a particularly pressing challenge to it. Determinism—although many definitions of this concept have been proposed—can be taken to state that the initial conditions of the world and the laws of physics influence every single state of the universe at every subsequent instant, including therefore everything related to the human being as a physical entity. If determinism is true, human beings can be equated with pool balls, or with the victims of an evil surgeon who manipulates our brain states to produce our choices and our actions (Vihvelin, 2003/2017; Cashmore, 2010). Historically, an answer to this challenge has been offered by compatibilism, which affirms the existence of a certain type of free will *in spite of* determinism.

If compatibilists are happy with the choice being freely caused by one's conscious desires (while desires might be determined by the law of physics), this response, which draws on a relevant and large philosophical tradition, has not always been considered satisfactory, except for pragmatic reasons. However, recent developments in the research on the interpretation of determinism and physical causation appear to reduce the scope of the determinist challenge. Ismael has, for example, made a convincing attempt at showing “how microlaws create the space for emergent systems with robust capabilities for self-governance,” arguing against the “threats to freedom that come from notions of causal necessity that physics has outgrown” (Ismael, 2016). The main idea is that “global laws do not imply strict necessity, nor do they impose a specific path on the universe given its initial conditions. This is because global laws have neither temporal asymmetry nor direction of influence. And the causal direction is given by modifying a variable in a subsystem that causes changes in another variable, within a framework in which there is a choice between exogenous and endogenous variables” (Lavazza, 2017b).

This does not mean that the challenge of determinism is outdated, but that today there are other threats to the traditional idea of free will that are more pressing and, apparently, more scientifically grounded, because they are not based on general laws but on the specific functioning of the mind/brain. Here we can distinguish—at least in general terms, because the levels are not clearly distinguishable—between the arguments that refer to metaphysical explanations and arguments that refer to epistemological explanations. If classic determinism if a genuine metaphysical claim, epiphenomenalism is related to psychological functioning of human beings and the interpretation of empirical data.

Epiphenomenalism is the thesis that seemingly causally relevant conscious processes, such as intention formation or decisions, do not play any active causal role in the production of the correspondent action. In general, the scientific arguments for epiphenomenalism start from the shared idea that free will implies a causal role of conscious mental processes. From this perspective, on the one hand, conscious mental processes should be explained in terms of scientific naturalism (which sets science as the sole measure of what exists and as the only method of knowledge) and this has turned out to be extremely difficult; on the other hand, in any case—most, if not all—our choices and decisions are taken to be guided by unconscious processes.

Following the useful clarification drawn by Nahmias (2014), even if it is objected that conscious mental processes can be naturalized as supervenient on underlying neuronal processes, the deflationary scientific perspective can answer the two following strategies. On the one hand, it can state, based on conceptual arguments, that the real causation is carried out by neuronal processes, and that conscious mental processes are only epiphenomenal. On the other hand, it can support, on the basis of empirical evidence, that the neuronal processes underlying conscious mental processes are not correctly “hooked” to the causal processes that bring about behavior, because, for example, they come too late (as in Libet's experiments), or in the wrong place (as in Wegner's experiments).

Nahmias calls the first scenario *metaphysical epiphenomenalism*. Like determinism and naturalism, it is relative to the form of causation; therefore, it is informed and affected only indirectly by the discoveries of cognitive sciences. In fact, all these theoretical positions are based on the general truth of knowledge about nature and of the brain in particular, but do not refer to single laws or explanations of cerebral functioning. Nahmias calls the second scenario *modular epiphenomenalism*. According to it, modules (a shorthand for somewhat encapsulated cognitive systems or processes) involved in conscious decisions or intention formation do not produce one's behavior, which instead is produced by modules that do not involve conscious states.

I will address this second form of epiphenomenalism, trying to show that it is not a knock-out argument against free will. I will not address the challenge of metaphysical epiphenomenalism instead. Surely, this is a major challenge to free will in purely philosophical-conceptual terms. According to Kim's exclusion argument (Kim, 1998), if our conscious mental states have no causal power, how can they guide our choices and our decisions based on a conscious reflection that answers to reasons? But upon closer inspection, one might maintain that not even the exclusion argument seems to have the final word on mental causation—let alone on free will (cf. Giorgi and Lavazza, 2018).

Since this article focuses in particular on the form of epiphenomenalism which implies that our choices are only consequences of external factors affecting our decision-making processes, it is useful to frame the rise of epiphenomenalism and its arguments both historically and conceptually. I will then try to show why both the empirical data and the arguments drawn therefrom do not seem sufficient to support the conclusion that our freedom is completely illusory.

## Free Will and Empirical Psychology

In order to clarify and address the challenge of epiphenomenalism to free will, I'll now very briefly retrace the history of the scientific research on the mind, from the perspective of the debate on free will. In my understanding, empirical psychology is part of the cognitive sciences (another view, for example, might take educational psychology to use empirical methods but not to be subsumable under cognitive sciences), which also include cognitive neuroscience. I will seek to highlight some core points that have led those who study free will to read the new experimental data as a basis to describe human behavior in terms of non-awareness and substantial automaticity. The premise is that the cognitive science studies conducted in the laboratory did not deal directly and specifically with free will, at least until Libet (Libet et al., 1983), and even after Libet they have mainly followed in his footsteps, so to speak (Saigle et al., 2018).

The basic assumption of classical cognitive sciences, of course, placed special emphasis on cognition, i.e., on all those conscious processes that contribute to making the agent aware of their environment and situation, evaluating their behavioral alternatives and deciding on the basis of intentions that may be the result of more general purposes, either given or consciously chosen at the time. This does not mean that classical cognitive sciences—with their representational-computational theory of mind—followed the general framework of intentional or folk psychology. Rather, they corrected the latter in many respects. Contemporary empirical psychology, which is fully part of the cognitive sciences, has helped to highlight how the so-called cognitive unconscious is not only an evolutively functional mode of action but also reflects an architecture of mind organized in modules with closed and automatic functioning. This acquisition has been inserted into more general views of the functioning of mind, for example the one elaborated by Fodor (1983, 2001), which alongside modularity also claims there is a central top-down processing that presides over the central functions and, from the perspective that interests us here, over the most relevant choices for the agent.

Another relevant strand is that which describes our mental architecture, and its consequent functioning, as fundamentally bipartite (Kahneman, 2011). According to this view, there are two mental/cerebral systems that divide cognitive work and often operate in competition. One is quick and automatic—automatic precisely in order to be quick—and substantially unconscious. It allows us to manage environmental situations that require reaction speed according to established behavior patterns and is probably the result of an evolutionary-adaptive path. The other system is slower, fully conscious and the result of a processing that also considers new and more functional behavioral schemes to respond to the environment. It goes without saying that in this framework conscious control is ensured by the “slow system,” whereas when the “fast system” takes over, our choices and actions tend to lose the typical characteristics of free choices and actions.

More recently, the most important development in the field of so-called new cognitive sciences has been the replacement of the “computer metaphor” with the perspective of embodied cognition: a set of theoretical proposals (on a broad experimental basis) united by idea that most of higher cognitive processes occur through the control systems of the agent body (or, in neuroscientific terms, of the motor brain), with the related limits and potentialities (Shapiro, 2010). The dynamic and embodied models, in the most radical theories, give up the representations considered neither really existent nor useful to postulate from the heuristic points of view (Chemero, 2009), canceling the distinction between subject and environment and introducing a single dynamic system (Port and van Gelder, 1995).

In this sense, the brain is considered a dynamic system in which the activity of the different neuronal populations (more or less active over time) synchronizes on different frequency bands that can operate in parallel or enter into competition. It is argued that cognitive processes such as attention, preparation and facilitation arise from phase synchronization between different frequency bands or phase-resetting phenomena in some frequency bands based on specific stimuli (Caruana and Borghi, 2016). For example, this would explain the top-down control of non-hierarchical type: in this case the attentive processes are not explained by a hierarchical structure of upper and lower areas but in terms of local self-organized phenomena.

The various oscillatory frequencies give rise to transient states that each have a different response to a stimulus of the same type and intensity. When, for example, there is a motor behavior, the stimulus is processed differently according to the oscillatory phase of the brain in which it is received. Consider a go signal (like a traffic light): according to the phase of the alpha rhythm in which this signal arrives, the beginning of the movement and the reaction times vary. This indicates that motor behavior must be interpreted within a situation of changing equilibrium that reflects multiple dimensions of the internal situation of the brain immediately preceding it. It should be noted that these are intra-individual variations in response that are detectable in an instrumental way and do not determine a significant effect except in particular situations (the reaction time at the start of a professional sprinter may vary from race to race by thousandths of a second). In other words, this idea of the brain as a dynamic system—if confirmed—may enrich our knowledge but does not seem to directly affect our concept of free will in a deflationary sense. Rather, it appears to trace brain functioning back to schemes that are more compatible with our idea of free will, like Churchland and Suhler's view of subcortical control (see section Dealing With Situationism).

What happens with motor behaviors also happens with sensory stimuli. When dealing with the borders of the human perception threshold, for example by administering a minimal electric current to the tip of a finger such that it is perceived at least in half of repeated administrations, the perception capacity depends strongly on spontaneous increases of activation in particular rhythms of oscillation of some cerebral cortices (Buzsáki, 2006). This sensory input may or may not be perceived, therefore, based on the immediately preceding transient status of a large-scale cortical network. It can be concluded that the external stimulus cannot be considered the only initial condition to evoke an answer: there is always a relationship with, and a reference to, the history of the cerebral state. Each cerebral state depends on the previous one, which has in turn interacted with external stimuli, in a dynamic chain which, however, seems to show certain consistency and continuity in the eyes of an external observer. This seems to mean that there is not a purely stochastic outcome of internal processes, but a repertoire that is built over time and which is drawn from every time.

In relation to embodied cognition, an interesting aspect is that of affordances, namely the dynamic relationships that are established between an agent and a perceived object, i.e., the opportunities for interaction with the physical entity that the subject deems achievable based on his own abilities and capacities (both physical and cognitive). Cisek (2007) proposed a model for the functioning of the motor system called “affordance competition hypothesis.” Our perceptive world generally manifests itself by offering us multiple possibilities for action. According to classical cognitive science, in a similar situation, first the brain selects the action to be performed and then plans how to do it in its motor details. Cisek's hypothesis (based on experiments) says instead that the brain processes several potential actions in parallel. These action plans compete with each other to be realized, trying to inhibit one another (in a subpersonal process that does not involve higher circuits nor the subject's awareness). In the end, albeit very quickly, various factors channeled to the prefrontal cortex lead to a decision in favor of a single action plan.

In relation to affordances are there real automatisms, as some pioneering studies in the area of embodied cognition seemed to show (Ellis and Tucker, 2000)? For example, on the basis of motor compatibility it seems that we are better and quicker at categorizing small objects if we have to press a small key and categorizing large objects if we have to press a large key, however we can consciously strive to improve our performances. But research in this field does not allow us to generalize these results. We are not driven by automatic processes related to unconscious body cognition, and the activation of affordances is modulated by goals and objectives through a top-down processing performed by the higher cognitive areas (Caruana and Borghi, 2016). Differences in categorization-performance with respect to congruence (small-small, big-big) exist and are a point in favor of embodied cognition, but they are not such as to question free will in areas that are relevant to the present discussion. This is because these phenomena only concern a part, though important, of our cognitive functioning, but not its totality.

On the other hand, there are experiments in which priming effects (behaviors triggered by clues or environmental elements, of which we are not aware at all or at least as causes of our behavior) or frail control effects seem to take over even in real life situations, restricting the scope of free will. For example, take a study that is often cited as an exemplary case of unconscious influence of the context on human behavior, which however encountered strong problems of replication (see section Dealing With Situationism). A group of American university students have been recruited for an unspecified psychological study. They were given a set of words with which to compose meaningful sentences, including numerous terms that, both in general and in American culture in particular, are related to stereotypes about the elderly, such as *wrinkles, gray, Florida*. Instead, a control group was given words containing neutral expressions with respect to age, such as *thirsty, clean, private*. At the end of the test, a monitoring system was set up in the corridor leading from the hall to the elevator: young people who had read and used the words connected to old age were walking more slowly compared to those who had read and used words unrelated to the later phase of life (Bargh et al., 1996). One may slow down one's pace because one's feet are sore or because one is trying to casually meet the cute person one saw come out of class the other day; however, it is bizarre to learn that one can walk slowly because one has just dealt with the words *wrinkles* and *Florida*. Therefore, it is reasonable to assume that our mind (or our brain) often, but not necessarily always, works and makes decisions by itself, without our conscious deliberation (in the sense of full awareness of a choice) (Wilson, 2004).

## The Epiphenomenalist Challenge to Free Will

As said above, epiphenomenalism claims that seemingly causally relevant conscious processes, such as intention formation or decisions, do not play any active causal role in the production of the correspondent action. Two influential strands of research that go in this direction are those inaugurated, respectively, by Libet and by Wegner.

As known, Benjamin Libet's experiments have been a huge contribution to the epiphenomenal idea of free will (Libet et al., 1983). According to those who interpret them in a deflationary sense about free will, such experiments indicate that participants do not take conscious decisions, but decide unconsciously and only become aware of their decision when the action has already begun at the level of the nervous system. The possibility of generalizing these findings, which however have been replicated with different results (Saigle et al., 2018), has led many scholars to consider these experiments as the evidence that most, if not all, of our decisions are taken unconsciously. The premise is that if an action does not come from a conscious decision-making process, it cannot be free.

The soundness of Libet's experiments can be challenged in many different ways (Lavazza and De Caro, 2010; Mele, 2014, chapter 2). First of all there is a controversial interpretation of the moment in which the decision is taken to perform the action relevant in experiments like Libet's (the flexion of the wrist, the pressure of a button). Does it happen when one agrees to participate in the experiment, or when the series of repetitions begins? Or does it happen exactly when it is detected by electroencephalography and electromyography? Some clues might suggest that the proximal decision actually occurs after the moment estimated in the experiments, bringing it close to the moment of its conscious perception (Mele, 2014, chapter 2). One can also think that the non-conscious brain activity that is thought to cause the decisions is actually only a portion of the conscious process that leads to intention or decision, or even a precondition of neuronal activation to make the decision (cf. Tortosa-Molina and Davis, 2018).

Recently, a series of experiments has radically questioned whether the readiness potential measured in Libet's experiments coincides with the causal input of decision making. These experiments seem to point to a different interpretation of the readiness potential, namely that the apparent build-up of the brain activity preceding subjectively spontaneous voluntary movements (SVM) “may reflect the ebb and flow of the background neuronal noise, rather than the outcome of a specific neural event corresponding to a ‘decision’ to initiate movement” (Schurger et al., 2016). And such brain activity is triggered by many factors, where “a computational model of decision making” is active and “sensory evidence and internal noise (both in the form of neural activity) are integrated over time by one or more decision neurons until a fixed threshold firing rate is reached, at which the animal issues a motor response” (cf. Schurger et al., 2012).

From the point of view of motivations, then, the choice of the moment in which to flex the wrist does not seem to have much relevance. It is an indifferent choice for the subject, to which she does not pay attention and for which she does not follow a line of reasoning and can, therefore, be taken almost automatically. Things are very different when it comes to important existential choices, which require a lot of thinking along with the utmost attention and awareness. Finally, it could be argued that a small “gap” in our awareness does not question the fact that we are endowed with free will. In fact, if the choice is taken consciously on the basis of a reason and our action follows from it, we can feel free even if at the cerebral level there is a small gap of consciousness between the decision making and the awareness of the action's beginning (Mele, 2014, p. 24–25).

This type of criticism can also be partly applied to experiments that have followed and refined Libet's ones (see Fried et al., 2011). In particular, Soon et al. (2008, 2013) used functional magnetic resonance imaging to predict with a success rate of about 60% what choices would be made by the participants before the latter became aware of them while putting them into action. Once again, these were not salient choices for the individuals nor can they be generalized to apply to all kinds of decisions, but in this case the research was designed to eliminate some of the confounding factors present in Libet's experiments. The difficulty in repeating this type of study in real life situations and the forecast rate still very far from 100% leave ample margin to support that such experiments do not provide a definitive demonstration of the epiphenomenal character of our choices and decisions, i.e., of the fact that they are accomplished in an unconscious way, guided by cerebral processes to which we have no direct access. One of the key points is that many of our decisions can be “distributed” over time and it is difficult to pinpoint the proximal choice that precedes the action.

On the other hand, a series of empirical psychology studies seem to support the idea that well-considered conscious decisions have a documentable effectiveness. Gollwitzer has developed a strand of research around implementation intentions, meaning the intentions of doing something in a specific place and time or in a specific situation. Some of the best-known examples concern the commitment to perform a breast self-examination in the next month. Dividing a sample of women into two groups, 100% of those who were asked to think about when and where they would do the examination and to write down their choice in a notebook did actually perform it, in the chosen time and places. In the other group, which had not been asked to think about the time and place of the examination, only 53% of women performed it. In another experiment, two groups of people who had just recovered from addiction to psychotropic substances had to write their resume to find a job. The first group was asked to think about when and where they would write their resume that day, while the second group was asked to think about when and where they would have lunch. The result was that 80% of the first group wrote the resume and no one in the second group did (Gollwitzer, 1999; cf. Gollwitzer and Sheeran, 2006).

These data also help reduce the scope of the well-known studies carried out by Wegner (2002, 2003). Through a series of ingenious experiments, Wegner has in fact tried to show that the experience of will—which, in most cases, is adequately coupled with our decisions and thus gives us the “illusion” of being the authors of our actions—actually involves a different mental module from the real mechanisms of volition. According to Wegner, this means that conscious will is certainly a useful compass to understand our behavior in the world but has no causal power. Like a compass, indeed, it does not affect the ship's route, even though it can indicate the direction taken at any moment.

Wegner's experiments tend to show that in the circumstances considered, individuals are easily fooled, believing that they are the authors of an action that is actually performed by others, or performing an action that they did not consciously want to perform (for example at a seance, without realizing it, participants move the table that is supposed to be moved by the spirits invoked). However, it is not obvious that one should draw Wegner's conclusion (Wegner, 2002, p. 144), namely that the “behavior that occurs *with* a sense of will is somehow the odd case, an add-on to a more basic underlying system.” Provided that there is no definitive evidence in favor of either position, I tend to agree with Mele (2014, p. 51): “Wegner says that something he regards as *necessary* for free will never happens. And I'm saying that this necessary thing sometimes does happen—that conscious intentions (or their neural correlates) sometimes are among the causes of corresponding actions. (…) My claim (…) is much less bold (…). Which of us is on firmer ground here?”

It could be argued that such arguments based on induction are not conclusive and that, in the case of Wegner and Mele, one could overturn the burden of proof. But, as I will show, there are cases in which people are not prey to external circumstances and decide based on internally generated intentions in a conscious way. The illusionists could then reply to these observations that the reasons why people wish to change their behavior inevitably stem from unconscious motivations about how one wants to behave, all of which has evolved for fitness. But this objection opens up an endless backward path that is hardly sustainable, because not all people develop the same motivations starting from the repertoire of predispositions with which they were born. One can therefore ask in what way we have come to be the people we are, making those choices. And the answer seems to include both random elements and conscious choices of the subject.

## Situationism

Situationism can be considered a subset of epiphenomenalism and seems to be a very pressing challenge to the idea of free will. In fact, it does not appeal to complex conceptual arguments nor to controversial neuroscientific experiments, but to the simple structured observation of the ordinary behavior of people in contexts often close to those of real life. In general, situationism endorses a frail control hypothesis (Doris, 2002; Appiah, 2008) about human behavior: according to it, the latter is conditioned by external and situational factors which arouse a response in us without us realizing that such factors are relevant or that they affect our behavior. This means that we have very little conscious control over our behavior, which goes against the idea that we are endowed with free will. To use the more specific terms of the psychological investigation, our actions—according to situationism—are the result of “automatic” consequences of environmental factors and not the result of the voluntary control exercised by the agent on their behavior.

Our habits, character and goals, which we believe to be the reasons for our choices, are actually less important than the minor contingencies we find every day. In other words, external factors are the prevalent ones, to the detriment of internal factors linked to the agent, thereby reversing the classical conception of freedom as an endowment of the subject. Of course, the influence of external factors is mediated by transient internal states. As we shall see, if you help someone after winning the lottery, this is most likely due to your good mood rather than a conscious choice.

Experiments on help behaviors have developed greatly since the 1960s (see Doris, 2002). For example, some participants were made to find a coin in a made-up phone booth, while others found nothing. Both groups could then choose whether to help a person gather some papers fallen out of a folder. The first group tended to help (87% of cases), while the second tended not to (only 4% of cases) (Isen and Levin, 1972). Another experiment was set in a mall: those who were asked to exchange a dollar banknote were much happier to do so when they could smell freshly baked bread or croissants, compared to those who did not (Baron, 1997). Another famous example is that of extreme obedience to authority in contexts that stimulate conformism, as in Milgram's (1969) experiment: here, participants were asked to administer electric shocks to patients in what they believed was an important scientific experiment (although the fact that 35% of the participants refused to take part in the ethically problematic phases of the experiment is often underestimated, cf. Racine and Dubljević, 2017). In all these cases, it is assumed that behavior is affected by the situation, subverting the predictions based on the person's character. Moreover, the volunteers involved in this kind of experiments tend to reject the explanation of their behavior in terms of causes they were not aware of, and instead motivate their choices with different reasons, made up to make their current conduct coherent with their general guidelines. In other words, the subjects refuse to accept the real motivations of their behavior as *justifications* for it.

The mechanisms underlying situationism fall at least partly within the broader category of non-conscious determinants of action and preferences, described as consequences of the automaticity of decision-making processes and of human action. According to Kihlstrom (2008), automated processes are characterized by: (1) *inevitable evocation*, that is, specific environmental stimuli give rise to specific responses, whatever the previous mental state of the subject involved; (2) *Incorrigible completion*, that is, once the automatic processes are triggered, they are carried out according to a defined scheme on which the subject cannot intervene; (3) *efficient execution*, that is, automatic processes do not require the subject's effort or active participation; (4) *parallel processing*, that is, automatic processes do not interfere with other simultaneous processes, nor interfered by them. An extreme theoretical version of the idea of pervasive automaticity was offered by G. Strawson. According to him, in short, for mental activities, and thought in particular, to count as mental actions, the agent must be able to voluntarily and consciously raise a content of thought. But in fact we cannot form the intention to think a specific thought: to do so, we should already have that thought available for consideration and adoption; and thought seem to come about automatically (Strawson, 2003). However, this is an indirect critique of the idea of free will, which is not strictly linked to empirical psychology and should be discussed at the philosophical level.

## Dealing With Situationism

Situationism has certainly improved the knowledge of the motives of human actions. In the light of increasing experimental evidence, it would be an unrealistic claim to think that people are not at all influenced by the circumstances in which they find themselves. Everything contributes and has a weight, but it is necessary to assess the relative importance of different factors, both internal and external to the individual. The main question is whether at times, when it comes to relevant choices, people can exercise their conscious control and act according to their own free will. In this sense, note that it has always been thought that the character of a person is identifiable and recognizable. Now, the only reliable, though impressionistic and non-scientific, way of inferring a person's character is to observe their behavior and choices so as to find some regularities. If we can identify someone's character, this means that there is a certain regularity (and predictability) in their behavior. As a result, it seems that this agent does not decide (only) on the basis of changing external circumstances, but on the basis of internal processes (their character) that are fairly stable.

Of course, even if human beings are very good at navigating their social environment on the basis of intentional psychology, they can still be the victims of cognitive biases and generally tend to categorize by amplifying differences and underestimating less salient aspects. To overcome this problem, psychologists themselves have constructed personality profiles to scientifically measure the constant behavioral orientations of individuals. While it is true that the existence of personality traits is controversial, and most personality tests have often been accused of being inaccurate, today we are making great progress in this direction thanks to big data. Gerlach et al. (2018), for example, have developed an alternative approach to the identification of personality types, applied “to four large data sets comprising more than 1.5 million participants.” The authors have identified four robust personality clusters by drawing a map of well-established personality traits—average personalities, reserved personalities; role model personalities, and self-centered personalities. And they also found that personalities develop and evolve, in general from “self-centered” in teenage years to other clusters in adulthood.

In any case, the idea of character as a stable tendency to react in coherent (if not predictable) ways to specific situations has now been affirmed, and the relevance of internal processes over external contingencies cannot be denied. In fact, the success of character-based explanations and predictions could otherwise only be explained by a very unlikely coincidence, by which random circumstances go mostly in the same causal direction as the agent's behavior. Situationists may argue that often one's character is not predictive (as their experiments show) and that personality profiles are not so reliable (Doris, 2002).

It is certainly true that often we make generic judgments, perhaps even biased by prejudices toward given social “categories,” due to which we unconsciously select observational data. But it's not always like that. For example, it has been noted that the presence of a large number of “righteous” people who risked their lives without hope of any reward, so as to save Jews threatened by Nazis, seems to disprove the situationist thesis (Fogelman, 1994; Monroe, 1996; Oliner and Oliner, 1998; cf. Ogien, 2001). Those people were not influenced by the situations in which they found themselves—which indeed would have led them to be accomplices or inert spectators, as many other people in that period. Instead, they showed coherence of character and personality over variable circumstances. Compassionate and courageous people of that kind seem to be a major problem for strong situationism, even if its supporters remain convinced that situationism can respond to this objection (cf. Machery, 2010).

Moreover, the surprising nature of the studies that highlight the role of environmental factors makes us underestimate that often most subjects—but not all—manifest the situation effect. Therefore, in general, the empirical basis cannot be used to affirm that the internal processes of the subject, supposedly underlying free will, are never at work. Another aspect concerns the fact that choices set up in laboratory experiments are not always relevant or typical of real life, and therefore it is more plausible that they may be influenced by contextual factors. This is not true, however, for the best known experiments. Consider, for example, the famous study showing how the participants' degree of altruism (the participants being seminarists) varied based on whether they were or weren't in a hurry due to some important commitment (Darley and Batson, 1973).

On a different level, we cannot fail to mention the issue of the reproducibility of social-behavior findings published in peer-reviewed journals (Open Science Collaboration, 2015). Failure in replication and problems with statistical processing have been detected for some time (Bakker and Wicherts, 2011), as a result of which 15% of studies have been reversed in their conclusions. And other studies also indicate that the arbitrary choices made by researchers in their study can increase false positives (Simmons et al., 2011). Even 38% of the articles published in *Science* and *Nature* could not be reproduced (Camerer et al., 2018). Interestingly, the authors of the latter study have created a prediction market, assembling a panel of about 80 psychologists and economists. They read the study and could exchange “shares” in the reliability of the result. The experts' “bets” went along with the overall replication rate, which shows that professional scholars are generally good judges of the empirical reality of the world. Therefore, if they “fail” certain very counterintuitive results, this might not mean that such results are normal and yet somehow went hitherto unnoticed, but rather that they are the outcome of exceptional conditions created in the experiment (type of environment, choice of participants…), obviously excluding the negligence and fraud of the researchers who conducted the unrepeatable experiment.

This is linked to another aspect of the experiments that gave rise to situationism: namely, the fact that they take place “below threshold” with respect to the social macro-interactions relevant to the dynamics of attribution of responsibility and to the functioning of interpersonal relationships. One could compare the relationship between the description of unconscious subpersonal mental mechanisms and intentional psychology with that between relativistic mechanics and classical mechanics. Relativistic mechanics is certainly more correct to the current state of knowledge and allows for a more “true” and finer description of reality, but the more intuitive and customary description offered by Newton with classical mechanics is perfectly adequate for many of the macroscopic applications that may concern us. When it comes to the description of the human being, moreover, there is also a subjective element, which might lead to prefer, for many reasons, the use of common sense in some areas of psychology.

It could also be said that what allows empirical psychology to describe the disunity of the subject and the automaticity of behavior is a “quantification” that covers a narrow area of our spectrum of social action. At the bodily level, we can measure the glycemic level of a subject and identify limits above and below which performance usually decreases and the state of health declines. The same applies to environmental parameters such as atmospheric temperature or the amount of oxygen. But even if we can follow the numerical parameters at all times, individual subjective states may vary compared to the recorded data, so that an individual may remain active even with a low reserve of sugars and under oppressive heat. Conversely, under formally ideal conditions, others may suffer from the cold or have a deficiency of organic resources. In other words, there is a central range of values for those parameters, so that only a major shift to either extreme significantly influences macroscopic behaviors. The same may hold for the fine effects detected in experiments in which people do not seem (and probably are not) fully free, conscious and rational in their choices. Significant interpersonal and social interactions could fall within that central macroscopic range of values of relevant parameters in which behavior is approximately free, conscious, and rational.

On the other hand, the acquisitions of situationism can also be considered a useful cognitive tool in order to make our behaviors less exposed to contingencies and more consistent with our deep motivations. This can happen, for example, in the case of the previously quoted seminarist experiment. Knowing that being in a hurry or even late for an important commitment (a pilot being expected at the airport) prevents us from acknowledging the urgent needs of others should induce people who want to be sensitive to the needs of others to leave home earlier, so as not to ignore any requests for help. Some studies tell us that this awareness is indeed in place (Beaman et al., 1978; Pietromonaco and Nisbett, 1982).

Finally, there is a line of research that, while taking seriously the non-conscious functioning of our brain, sees it as the result of conscious learning process, according to an Aristotelian approach revised in the light of new neuroscientific knowledge (Suhler and Churchland, 2009; Churchland and Suhler, 2014). In particular, the proponents argue that the reward system, which has so much weight in our choices, is part of us, even if it acts in a mainly automatic way; it can be educated and receives continuous feedbacks. Our choices are authentic, coming from inside and not as the effect of external circumstances, because we are our brain. To the situationists' experiments, these scholars object that “matters looks very different when you balance the picture with scientific data showing the robustness of control, such as the capacity to maintain a goal despite distractions, to defer gratification, to stop an action midway, to develop advantageous habits, and to suppress impulses. This is seen in human, but also in monkeys, rats, and, one has to predict, in many other species” (Churchland and Suhler, 2014, p. 314–315).

The emphasis is on “learned industriousness,” which might indicate the role of the reward system in reinforcing behavior patterns that cause persistence in pursuing a goal (Eisenberger et al., 1992). According to advocates of this perspective, through the reward system, the very feeling of intense and prolonged effort can become rewarding in itself. This observation of neuronal activations indicates both a robust ability to control and the fact that such ability can be strengthened through reinforcement. In the Aristotelian sense, if we cultivate and incorporate a second nature, even if our choices and decisions are “automatic” they will be a “free” expression of what we want to be.

## A Realistic Free Will in the Light of Epiphenomenalism

Certainly, self-control and free will do not only depend on conscious processes. But, in the context of the global workspace account, which is now accepted by many as regards personal-level access to the information content of mind/brain states, consciousness plays the role of integrating the contents of consuming systems. According to Levy, the global workspace model implies that consciousness makes a difference to our choices, even if non-conscious mental states also influence our behavior. “There are systematic differences in how these states influence behavior with and without consciousness, and these differences entail a difference in our degree of control over certain facts. Only when information is integrated does the *agent* exercise control over the extent to which that information influences his or her behavior” (Levy, 2014, p. 336).

Levy's examples on free will include the observation made by Penfield (1975), according to which patients affected by an epileptic attack follow a habitual and stereotyped pattern of behavior but lose the ability to make decisions with respect to situations that they have never encountered before. This inability can be explained by the impossibility of (consciously) accessing a wide range of information, while in turn explaining the rigidity of behavior during epileptic attacks. The famous judicial case of somnambulistic violence (Broughton et al., 1994), in which the perpetrator of a crime committed while sleepwalking was acquitted, can be seen in the same neurological terms. An otherwise perfectly healthy person got out of bed in his sleep and went to the house of his parents-in-law and stabbed them, without ever leaving the sleepwalking state, even though the two victims were screaming and tried to defend themselves. The subject was in a situation where he did not understand the contradiction between his beliefs and values on the one hand and his behavior on the other. The actions of the subject in that altered state of consciousness were not expressive of, nor controlled by, a sufficiently broad spectrum of his attitudes, given that those attitudes made him the person he used to be. Unaltered consciousness, in fact, gives control to the agent as a whole by integrating all the information available.

Only consciousness in its normal functioning allows for access to, and the evaluation of, not only the perceptive inputs but also the motivations, beliefs and values of the subject, in the process that is typically associated to free will. In this sense, the idea that there must be conscious choices for behavior to be considered free has not only a philosophical value but refers to the effective functioning of our brain. For example, the acquisition of new skills requires the participation of areas associated to the global workspace, in particular large areas of the cortex, but once the new skills are acquired the areas that are activated by their use are greatly reduced (Haier et al., 1992; Raichle et al., 1994). An action that involves the use of those skills can be considered free (though not necessarily) even in subsequent situations because the agent had previously consciously acquired them.

To the present state of knowledge, all this appears to be true. However, this does not mean that all the evidence supporting modular epiphenomenalism, despite its limits, can be ignored. Such evidence does not deny free will for the factual and conceptual reasons outlined so far, but it does not leave things as they were before situationism either. Taking up the conditions of free will exposed at the beginning, many philosophers support what can be called reasons accounts of free will (Wolf, 1990; Wallace, 1994; Fischer and Ravizza, 1998; Arpaly, 2003). Based on these accounts, the ability of the agent to respond appropriately to reasons is what gives the subject the control typical of free will (and necessary for moral responsibility). The reasons accounts have many points in their favor, starting from the adherence to the intuitive idea of free will. But they are also the ones that are most often challenged by situationism, as situationism *prima facie* shows a degree of irrationality in our behavior or at least a rationality that is too low to be able to affirm that we have free will.

Many objections and criticisms can be made to the general argument of situationism, that is, that we do not enjoy free will as it is classically understood. However, as Vargas(2013, p. 333) usefully noted, much and robust empirical evidence indicates that “our rational, moral natures are very fragile and bounded.” For example, this is shown by an experiment like the following. Two groups of students are subjected to a test in which they have to underline the pronouns used in the report of a school trip (*us, ours, me, my*). Those who read the passage with the plural pronouns are more likely to indicate as “guiding principles of one's life” relational values (such as *belonging, friendship, security, family*) compared to those who have read the text “in the singular” (Gardner et al., 1999, 2002).

In the light of all this, Vargas suggests we recast reasons accounts and give up some of the suppositions that are usually implied by such accounts. The first is *atomism*: “the view that free will is a non-relational property of agents; it is characterizable in isolation from broader social and physical contexts.” The second is *monism*: “the view that is only one natural power or arrangement of agential features that constitutes free will or the control condition.” Given the “situation-dependent nature of our capacities”—or, as I prefer to say, the “relational nature of our capacities' implementation”—one can embrace a pluralistic account, which holds “that there are multiple agential structures or combinations of powers that constitute the control or freedom required for moral responsibility” (Vargas, 2013, p. 333). In other words, faced with the variability of our ability to control our actions, even depending on the external situation in which we find ourselves, we can moderately reconsider the idea that “our capacities for control are metaphysically robust, unified, and cross-situationally stable” (Vargas, 2013, p. 341).

## Conclusion

The concept of free will has generally been challenged on the metaphysical front by the apparent impossibility of jointly supporting the truth of determinism and the existence of freedom. In order to do so, compatibilism has been a widespread philosophical stance on this topic. Advances in psychological and neuroscientific research have now shifted the challenge to free will from the metaphysical to the epistemological level. The most recent expression of this challenge goes under the name of epiphenomenalism, understood as the thesis according to which the subject's conscious decision-making guiding their behavior is only apparent.

A series of studies have focused on the brain mechanisms of action initiation and on the timing of consciousness, using brain-activity probing techniques. Another line of studies—recently called situationism—has instead investigated the unconscious influence of environmental stimuli and situations on the subject's behavior, which are capable of conditioning the subject's choices without them being aware of it. In my article, I showed that Libet's experiments and those that followed are not conclusive for various reasons and therefore do not call into question the idea of freedom, at least not in the situations, which cannot be tested with the current brain-imaging techniques, where the choice to be made is significant.

As for situationism, its challenge to free will seems to be more insidious. Even if the replicability of many studies is low or controversial, it does not seem possible to deny that priming effects are significantly at work, at least in some circumstances. The choices made under the implied push of environmental elements that we usually consider of little importance can hardly be defined as free according to the definitions proposed at the beginning of the paper. There are, however, numerous counter-examples to situationism. Furthermore, it is reasonable to assume that the subjects informed of the priming effect, or anyway educated of the risk of being conditioned by the environment, can increase the degree of freedom of their choices, even in cases where situationism would otherwise be effective.

Therefore, one can reasonably conclude that the data available are not sufficient to deny that we are endowed with free will in the form of conscious control that makes us morally responsible for what we do. Rather, there are enough data to say that we are *not always* free, and in any case not free in the same way every time we make a choice. In different situations, also based on our explicit and conscious effort, our degree of freedom can vary. Accordingly, one can think of free will as an operationalized concept, which comes in degrees and might be measured with proper tests and neuropsychological means, as I proposed elsewhere (Lavazza and Inglese, 2015).

## Author Contributions

The author confirms being the sole contributor of this work and has approved it for publication.

### Conflict of Interest Statement

The author declares that the research was conducted in the absence of any commercial or financial relationships that could be construed as a potential conflict of interest.
